# Prevalence of Pain in Patients with HIV/AIDS: A Cross-sectional Survey in a South Indian State

**DOI:** 10.4103/0973-1075.53550

**Published:** 2009

**Authors:** Shoba N Nair, Theophin Regina Mary, S Prarthana, Preethy Harrison

**Affiliations:** Department of Pain and Palliative Care, St. John's Medical College and Hospital, Bangalore - 560 034, India; 1 Department of Snehadan, Karmalaram Post, Bangalore - 560 035, India

**Keywords:** HIV/AIDS Patients, Pain, Prevalence

## Abstract

**Objectives:**

Primary - To measure the prevalence of pain in HIV/AIDS with patients.

Secondary - To assess the type, site, severity, management of pain and impact of pain on quality of life in these patients.

**Design:**

Multicentre cross-sectional survey (This paper is a pilot study).

**Settings:**

ART centre at St. John's Medical College Hospital, Bangalore and Snehadan, A supportive and care centre for HIV/ AIDS patients at Bangalore.

**Materials and Methods:**

Data sheet, Brief pain inventory and Short – Form McGill pain questionnaire.

**Subjects:**

This is an ongoing study and the pilot study includes 140 HIV/AIDS patients in different stages of the disease.

**Results:**

About 66.7% (28/42) in-patients and 24.5% (24/98) out-patients complained of pain. Of the 52 patients who reported pain, 32% (14/52) reported neuropathic pain and 68% (38/52) reported noci-ceptive pain. Headache was most common followed by pain in the soles of feet and low back. Only 26.9% (17/52) received any form of analgesic. Pain severity significantly affects the quality of life.

**Conclusions:**

Pain is a common and debilitating symptom of HIV/AIDS. It is however, under-estimated and under treated.

## INTRODUCTION

Pain is often called the fifth vital sign and can reveal a lot about the health status of a person. Pain also affects the quality of life through its effect on such things as mood, activity, appetite, sleep, hygiene and the ability to focus and concentrate. Pain is almost always under-estimated as it is subjective. One patient's severe pain would be moderate pain for another patient. Assessment of pain in a patient is very important.

It is more than a decade since pain has been acknowledged and studied in HIV/AIDS patients in different parts of the world. India has the second highest population of HIV/AIDS patients in the world, however, prevalence of pain in these patients has never been acknowledged. In HIV patients, pain is often underestimated, as the focus is on the disease itself. Á multi-centre study on under-estimation and under treatment of pain in HIV disease published in the BMJ in 1997[1] concluded that pain is a common and debilitating symptom of HIV disease which is gravely underestimated and treated. The analysis of this multicentre study showed that among 78 AIDS patients for whom a source of pain was identified, 26 (33%) was reported to have digestive or mouth pain, 25 (32%) muscular pain, and 16 (20%) joint or bone pain. Central nervous system pain was reported for 15 patients (19%) and painful peripheral neuropathies for 10 (13%). Prasithsirikul *et al.*,[2] in their International presentation on AIDS observed that in Thailand there was a high prevalence of pain in HIV patients which was being inadequately addressed. Del Borgo *et al*.,[3] in their article on pain in HIV patients say that pain is one of the most frequent symptoms in these patients and it is present at all stages of disease although it is more frequent in the advanced stages. In her article on pain and palliative care for people living with HIV/AIDS in Asia, Marie Coughlan[4] observed that the prevalence of pain in HIV/AIDS patients is as high if not higher than in cancer patients. As early as 1999 Bernard N *et al.*,[5] said the prevalence of pain in HIV patients is between 30-80% and pain is frequently under-estimated by physicians.

Pain experienced by HIV patients can be due to multiple sources: firstly, the HIV infection itself or its consequences (infections, tumors); secondly, treatments for AIDS; or, thirdly, it can be unrelated to the disease and its treatment.[1] HIV related pain has been found to impair both functional and affective components of daily life.

## MATERIALS AND METHODS

This study has been designed as a cross-sectional survey. As this is a pilot study, data from two centres is being presented here. HIV/ AIDS patients from ART centre at St. John's Medical college hospital and support and care centre ‘Snehadan,’ were recruited. The ART centre is an outpatient facility, whereas ‘Snehadan’ is for in-patients. All HIV positive patients, irrespective of their staging, have been included in this study. Patients who were already diagnosed with diabetic neuropathy, arthritis, gout, sciatica and trauma induced pain were, however, excluded.

A data sheet, brief pain Inventory and short–form McGill pain questionnaire on type of pain by Ronald Melzack was used in this study. The data sheet included patient demographics, WHO staging of the disease, CD4 count, ART or not, presence of pain, substance abuse, since diagnosis and the performance status of the patients. The Brief pain Inventory (BPI) has been used previously to measure pain in HIV/AIDS patients.[1] The BPI asks patients to report if they experienced pain because of their disease during the previous week and to rate their pain (worst, least and on an average) on a 0-10 numerical scales. The patients were also asked to rate their quality of life which assessed their general activity, mood, sleep, walking ability, working ability, relationship with others and enjoyment of life. They were explained about the study and data was collcted following an interview. An informed consent was taken from all patients. The principal investigator/one of the co- investigators conducted the interview in English. The interview was conducted in the patient's own language and each question was translated verbally and asked by the investigator. This is an observational study and descriptive statistics has been used.

## RESULTS

A total of 42 in-patients and 98 out-patients participated. Patients’ ages ranged from 18-68 yrs with a maximum number of patients in the 31-40 years age group (56). The sample consisted of 59% males and 41% females. Of the 140 patients, 87% were literate and 71% were employed. The number of patients in the first and second stages of the disease was equal to the number of patients in the third and fourth stages. Ninety percent of the patients were able to walk [Table 1].

**Table 1:** Demographics

Gender		Employment	
Males	81	Yes	100
Females	59	No	40
Age		Staging	
18-31 yrs	40	I	49
31-40 yrs	56	II	16
41-50 yrs	30	III	35
51-69 yrs	9	IV	38
Education		Missing	2
School	88	WAB	
College	33	Walking	127
Illiterate	19	Ambulatory	12
		Bed	1

### Pain

Pain was significantly more common for in-patients (66.7%) than for out-patients (24.5%). Almost 79% of patients who complained of pain were in stages III and IV of the disease, whereas 19.2% of stage I complained of pain. When we looked at the number of patients in these two stages of the disease amongst the in-patients and the out patients, 90% of the in-patients and 40% of the out-patients were in the third and fourth stages of the disease. Amongst patients who complained of pain, 60% were on ART. The site of the pain in these patients varied. The three major causes (sites) of pain were headache (28.75%), soles/leg pain (25%) and backache (19.23%) [Table 2].

**Table 2:** Pain-site

Headache	28.75
Soles/leg pain	25
Backache	19.23
Abdomen	7.60
Joint pain	7.60
Generalised ache	5.70
Mouth & vulval area	3.80
Perineal area	3.80
Chest pain	1.90

All values are in percentages

Least pain was reported by 73.08% of the patients as 0-3, by 23.08% as 4-6 and by 3.84% as 7-10 on the numerical scale. Average pain was reported by 50% of the patients as 1-3, by 48.07% as 4-6 and 1.92% as 7-10; where as worst pain was reported by 13.47% as 1-3, 42.30% as 4-6 and 44.23% as 7-10 on the numerical scale. When we looked at the type of pain almost 68% had noci-ceptive pain and 32% had neuropathic pain. People who described their pain as aching, cramping, splitting, tender or heavy, according to the McGill Short –Form Pain Questionnaire were categorized as having noci-ceptive pain and those who described their pain as shooting, stabbing, sharp or burning were categorized as having neuropathic pain. Headache can be grouped under noci-ceptive pain. Of the 28.75% of the patients who reported headaches, 25% were on ART. About 23.75% of the patients described pain in two sites and 11. 85% described pain in three sites.

### Analgesics and pain relief

Only 26.92% of patients received any analgesics (paracetamol, NSAIDs, anti convulsant, anti depressant). None of the patients received any form of opioids. Amongst the patients who received analgesics only 21.1% had 70% and more of pain relief. The rest of the patients did not have adequate pain relief.

### Quality of life

The brief pain inventory also looked at the quality of life of these patients. There was significant impairment in their quality of life. Around 73% of patients reported that the pain affected their general activity. Sixty seven percent reported that it affected their mood, while 63% reported that it affected their sleep. As high as 73% reported that the pain affected their working ability thereby reducing productivity.

## DISCUSSION

This survey is the first of its kind in India. Prevalence of pain is high in HIV patients. There is inadequate assessment of pain, inadequate management and inadequate pain relief for HIV/ AIDS patients with pain. The added effect of this is impaired quality of life and reduced productivity. As shown in other studies, in this study also there is higher prevalence of pain amongst the in-patients. This suggests that pain increases as the disease progresses.

Inadequate assessment of chronic pain leads to inadequate management and relief of pain. For example, if a patient reports pain, the doctors should be able to assess it's duration and frequency, what is causing the pain, what is the severity and what is the type of pain. Health care professionals should be able to differentiate between different types of pain so that optimum treatment can be given. We have not got out of our habits of prescribing analgesics on a PRN or SOS basis. For chronic pain analgesics should be prescribed round the clock and as long as it is required. Pain in HIV can be due to different opportunistic infections that these patients can have. Along with analgesics, we should never forget to look at the root cause of the pain and treat it accordingly.

None of the patients in our study received any form of opioids. This might not hold true if it is a multi-centric study with a big sample size. Most of the doctors, in the field of HIV/AIDS care, are not familiar with pain management. This is where Palliative care training can make a big difference to the quality of life of these patients.

### Limitations

One limitation noticed in the pilot study was that we had only looked at whether the patients were on ART or not. The actual drug that the patient was on was not looked at. Therefore the study could not co–relate headaches with zidovudine or peripheral neuropathy with stavudine. This needs to be rectified in the main study being planned at 40 different centers in Karnataka.
